# Fine-Tuning Intrinsic and Doped Hydrogenated Amorphous Silicon Thin-Film Anodes Deposited by PECVD to Enhance Capacity and Stability in Lithium-Ion Batteries

**DOI:** 10.3390/nano14020204

**Published:** 2024-01-17

**Authors:** Nieves González, Tomás García, Carmen Morant, Rocío Barrio

**Affiliations:** 1 Renewable Energy Division, CIEMAT, Avenida Complutense 40, CP-28040 Madrid, Spain; nieves.gonzalez@ciemat.es; 2 Department of Applied Physics, Autonomous University of Madrid, Calle Francisco Tomás y Valiente 7, CP-28049 Madrid, Spain; tomas.garciar@estudiante.uam.es (T.G.); c.morant@uam.es (C.M.); 3 Instituto de Ciencia de Materiales Nicolás Cabrera, CP-28049 Madrid, Spain

**Keywords:** anodes, lithium-ion battery, amorphous silicon, energy storage

## Abstract

Silicon is a promising alternative to graphite as an anode material in lithium-ion batteries, thanks to its high theoretical lithium storage capacity. Despite these high expectations, silicon anodes still face significant challenges, such as premature battery failure caused by huge volume changes during charge–discharge processes. To solve this drawback, using amorphous silicon as a thin film offers several advantages: its amorphous nature allows for better stress mitigation and it can be directly grown on current collectors for material savings and improved Li-ion diffusion. Furthermore, its conductivity is easily increased through doping during its growth. In this work, we focused on a comprehensive study of the influence of both electrical and structural properties of intrinsic and doped hydrogenated amorphous silicon (aSi:H) thin-film anodes on the specific capacity and stability of lithium-ion batteries. This study allows us to establish that hydrogen distribution in the aSi:H material plays a pivotal role in enhancing battery capacity and longevity, possibly masking the significance of the conductivity in the case of doped electrodes. Our findings show that we were able to achieve high initial specific capacities (3070 mAhg-1 at the 10th cycle), which can be retained at values higher than those of graphite for a significant number of cycles (>120 cycles), depending on the structural properties of the aSi:H films. To our knowledge, this is the first comprehensive study of the influence of these properties of thin films with different doping levels and hydrogen distributions on their optimization and use as anodes in lithium-ion batteries.

## 1. Introduction

Within the scope of energy storage, silicon has emerged as one of the most promising alternatives to replace graphite as an anode in lithium-ion batteries (LIBs) due to its high theoretical lithium storage capacity, which can be up to eleven times higher than that of graphite [1,2,3]. However, despite the high expectations for silicon-based anodes, commercial LIBs with these anodes still present limitations that must be addressed. During battery charging and discharging processes, crystalline silicon undergoes significant volume changes (over 300%) that degrade the material and ultimately cause it to fracture and detach from the metal collector, leading to failure and shortening the battery lifetime. Another drawback of silicon is its low electrical conductivity compared to graphite (1 S·cm^−1^), which limits its use in rapid discharge applications. Consequently, significant efforts are being made to address these disadvantages, resulting in numerous research reports that offer a range of solutions. Among these potential solutions, nanostructured crystalline silicon stands out, as it can be molded into structures such as nanorods, nanowires, nanospheres, etc., that are designed to absorb volume changes, preventing premature failure of the batteries [4,5,6,7].

In the current context, thin films composed of amorphous silicon offer several advantages over those made of crystalline silicon. Its amorphous nature enables it to effectively mitigate stresses arising from lithiation and delithiation processes. Additionally, hydrogenated amorphous silicon thin-films (aSi:H) can be directly grown on current collectors with excellent adhesion, eliminating the need for binders that may be necessary for crystalline silicon electrodes. Furthermore, the conductivity of amorphous silicon can be readily increased by introducing dopant elements into its lattice, thereby improving the performance of lithium-ion batteries (LIBs) with this type of anode [2]. The use of thin films with thicknesses of a few micrometers also facilitates the diffusion of lithium ions into the material [8], which enhances the electrochemical performance and also enables considerable material saving.

Plasma-Enhanced Chemical Vapor Deposition (PECVD) is one of the most commonly used techniques for depositing amorphous silicon thin-films. The versatility and possibilities provided by this PECVD technique are indisputable, making hydrogenated amorphous silicon a prominent material in the development of solar cells, including p-i-n type and highly efficient silicon heterojunction solar cells [9,10]. The use of hydrogenated amorphous silicon films extends beyond solar cells to include applications such as thin-film transistors for liquid crystal displays, semitransparent solar cells, flexible electronic devices, and electrodes in LIBs [11,12,13,14,15]. In this study, we investigated the influence of the electrical and structural properties of electrodes based on hydrogenated amorphous silicon films, deposited by PECVD (13.56 MHz), on the electrochemical properties of lithium-ion batteries. Specifically, we analyzed how the electrical conductivity and hydrogen content in the form of polyhydrides bonds (Si-H_x_) of aSi:H affect both the specific capacity and capacity retention of LIBs.

Our extensive experience in developing aSi:H thin films using PECVD for photovoltaic applications has been instrumental in this research [16]. In our previous works [5,6], we studied the preparation conditions of aSi:H in depth and used this material to generate amorphous silicon nanowires (a-SiNWs) with optimal lengths and density to be used as anodes in LIBs. Our findings revealed how small deviations in the hydrogen content and its arrangement in the material significantly affected the growth of a-SiNWs through a metal-assisted chemical etching process. In the work that we have now developed, we have gone further by characterizing intrinsic hydrogenated amorphous silicon non-nanostructured thin films deposited by PECVD under different conditions to determine their total hydrogen content, the hydrogen arrangement in the material lattice, and optical properties such as the refractive index. Subsequently, this material was directly grown on surface copper foils and then assembled in the form of electrodes in LIBs. The specific capacity and capacity retention of the as-prepared batteries were analyzed as functions of the aSi:H film properties. In addition, we prepared n-type doped aSi:H films with varying electrical conductivities and assessed how their electrical properties affected both their hydrogen composition and their specific performance in lithium-ion batteries. To the best of our knowledge, a comprehensive study of the role of conductivity and polyhydride concentration of intrinsic and n-doped aSi:H thin film-based anodes for LIBs has not yet been carried out.

## 2. Material and Methods

### 2.1. Preparation and Characterization of Hydrogenated Amorphous Silicon

The hydrogenated amorphous silicon obtained using PECVD, which is made from a gaseous mixture in which silane (SiH_4_) is the main component, is a highly hydrogenated material. Both the total hydrogen content and the way the hydrogen atoms are attached in the material lattice will deeply depend on the PECVD deposition conditions. The hydrogen can then be bonded as isolated Si-H bonds, called monohydrides, or it can form clusters of Si-H_x_ bonds, referred to as polyhydrides, which are mainly located in the inner walls of material microcavities. The relation between these forms of hydrogenation (Si-H_x_ vs. Si-H bonds) defines the aSi:H quality in terms of its mass density or porosity and the amount of defects in its bulk [17,18]. Therefore, the greater its polyhydride (Si-H_x_) concentration the more porous and defective the material will be [19,20].

The PECVD allows the preparation of thin films with good homogeneity at substrate temperatures below 250 °C. Furthermore, both the properties and the structure of the material and the thickness of the films can be easily tunneled by properly adjusting the deposition parameters. In addition, silicon alloys, for example, silicon carbides and silicon nitrides [16] and doped silicon with boron (p-type) or phosphorous (n-type) atoms, can be obtained by adding gaseous compounds with the required elements to the precursor silane gas. Therefore, this growth technique provides a wide variety of materials for a large number of applications. In this work, we have prepared both intrinsic and n-type doped hydrogenated amorphous thin silicon films. N-type doped films with variable electrical conductivities were deposited using a mixture of silane (SiH4, 99.999%) and phosphine (2% PH_3_ diluted in hydrogen gas, H_2_) in which the phosphine flow-rate was intentionally modified.

The thicknesses and refractive indexes at 633 nm (n_633_) for the deposited films in this study were determined optically using a UV/VIS/IR Lamba1050-Perkin Elmer spectrophotometer, in the range between 300 nm and 2500 nm, according to the procedure described in previous works [5,16]. The dark conductivity (σ) of the material was estimated based on the current–voltage characteristics (I-V) at room temperature, for which aluminum contacts were evaporated in a coplanar configuration on the films. Moreover, we evaluated the doping level of material by analyzing its activation energy (Ea), which was determined based on the conductivity versus temperature plots.

The hydrogen content and its bonding configuration in the material structure were examined. These parameters were determined based on the infrared absorption spectra of Si:H films deposited on high-resistivity monocrystalline silicon wafers by using a PerkinElmer Lamda 100FT-IR spectrophotometer, as we described in [16]. This technique allows us to obtain information about the microstructure of the material, analyzing the positions of the characteristic bands in the spectrum and their relative intensities. Thus, the overall hydrogen content of the material was determined based on a band located at 633 cm^−1^, which corresponded to the *rocking* and *wagging* vibration modes.Instead, the *stretching* mode bands (SM) centered at 2000 cm^−1^ and 2100 cm^−1^ describe the different bonding configurations of the hydrogen in the structure: the formation of isolated monohydride bonds (Si-H) for low-frequency modes (LSM, corresponding to FTIR band at 1980–2010 cm^−1^), and polyhydride bonds (Si-H_x_) arranged mainly on the inner walls of voids for high-frequency modes (HSM, corresponding to the FTIR band at 2020–2100 cm^−1^) [21,22]. Based on these FTIR measurements, we have defined the microstructure factor (R_SM_) as the ratio between the intensities corresponding to the low-frequency band LSM and the high-frequency band HSM (Equation (1)):

(1) $$R_{SM}=\frac{I_{HSM}}{I_{LSM}+I_{HSM}}$$

which indicates what proportion of hydrogen with respect to the total hydrogen content is incorporated into the structure of the material in the form of SiH_x_ bonds. R_SM_ is a qualitative method of evaluating the material in reference to its porosity and defect density. The higher R_SM_, the higher the number of polyhydrides, which corresponds to a material with a greater presence of nanovoids, less density and with a greater number of defects [19,23]. The analysis of the hydrogen content of the different types of bonds [H] was performed according to the method described in [24] with the corrected proportionality factors determined by Langford et al. in 635, 2000 and 2100 cm^−1^ [21].

### 2.2. Preparation of the aSi:H Electrodes, Assembly in Lithium-Ion Batteries and Electrochemical Characterization

**Figure 1:**
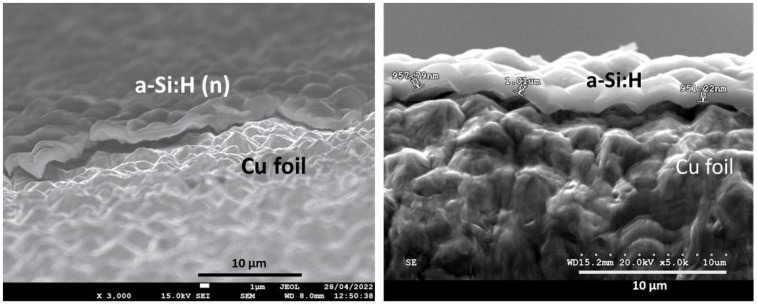
Cross section of FESEM images of a-Si:H thin films deposited on the rough side of Cu foils. The aSi:H thickness was approximately 1 µm.

A copper substrate (thickness = 9 μm, purity = 99.9%, MTI Corporation) was used to deposit the a-Si:H films. The Cu foil was pre-cleaned with ethanol and the rough side was chosen for the deposition in order to increase the adhesion of the deposited material. The thickness of the aSi:H(i) films was about 1 micron and strong adherence to the rough side of the Cu foil was achieved, as illustrated by the FESEM images in Figure 1. These electrodes were cut into 12 mm-diameter circles by a high precision punching EL-CELL. Then, the aSi:H active mass was determined by weighing the electrodes on a 0.01 mg high-precision balance and subtracting the average value of the Cu substrate mass obtained from ten identical circular Cu samples. The masses of the aSi:H films are significantly smaller compared to those of the Cu substrate. Additionally, the calculated standard deviation was approximately 13%, which is quite high, particularly given the low values of the masses. It is important to consider this value when interpreting the results obtained from the specific capacity curves.

After drying the electrodes in vacuum (90 °C, 10–3 mbar, 10 h), the batteries were assembled in a half-cell configuration, i.e., amorphous silicon was used as the cathode instead of Li metal (reference electrode), although in full-configuration commercial batteries, Si would be the anode. The Cu foil on which the aSi:H film was deposited acted as a positive current collector and a stainless-steel foil placed on top the Li electrode acted as a negative current collector. Battery assembly was performed inside a Jacomex Campus glovebox with Ar atmosphere (<1 ppm O_2_) in CR2032 button cells by using borosilicate glass fiber (Whatman GF/B) as separator and 1.0 M solution of lithium hexafluorophosphate (LiPF_6_) in ethylene carbonate and diethyl carbonate (50/50 (*v/v*)) as the electrolyte.

To evaluate their stability and capacity, the batteries were subjected to long cycling of 200 galvanostatic charge/discharge cycles at 1 Ag^−1^, by means of a potentiostat at room temperature (Arbin Instruments BR2143, 12 channels). The first two cycles were tested at low current density (200 mAg^−1^) to ensure the formation of a homogeneous solid-electrolyte interface (SEI). The specific capacitance (mAhg^−1^) presented in the Galvanostatic charge–discharge (GCD) curves was obtained as the product of current and cycle time divided by the active mass of the aSi:H material. Differential capacitance profiles (dQ/dV) were also extracted from the GCD curves for different cycles.

## 3. Results and Discussion

### 3.1. The Hydrogenated Amorphous Silicon Thin-Films’ Properties

The structure, composition and properties of aSi:H films deposited by PECVD are strongly dependent on the preparation conditions. Variations in PECVD parameters such as power, pressure and substrate temperature lead to more or less hydrogenated films with very different ratios between monohydride (Si-H) and polyhydride (Si-H_x_) bonds and, consequently, variable characteristics. In addition, we observed that incorporating dopant elements in the structure to improve the electrical properties causes a change in the composition of the material, increasing the overall hydrogen content and the polyhydride concentration. Substrate temperature (T_S_) is one of the deposition parameters that allows the intentional adjustment of the hydrogen bonding configuration [25,26,27]. For this reason, we have prepared several series of samples in which we exclusively changed the phosphine flow rate (Φ(PH_3_/H_2_) in the range of 0 to 15 sccm at different substrate temperatures between 140 °C and 250 °C in order to obtain intrinsic and n-doped films with different electrical properties (conductivity and activation energy) and hydrogen contents and distributions. The other preparation parameters were maintained unchanged (i.e., Φ(SiH_4_): 20 sccm; chamber pressure: 550 mTorr; power density: 18 mW/cm^2^). Using these deposition parameters, the optical, electrical and structural properties of amorphous silicon films have been analyzed as functions of the substrate temperature and the concentration of phosphine R, which is defined as the ratio between the flow rate of phosphine/hydrogen mixture and that of silane (Equation (2)). Then, some of these films were tested as anodes in LIBs in the next section.

(2) $$R=\frac{\Phi ({PH}_{3}/H_{2})}{\Phi ({SiH}_{4})}$$

**Figure 2:**
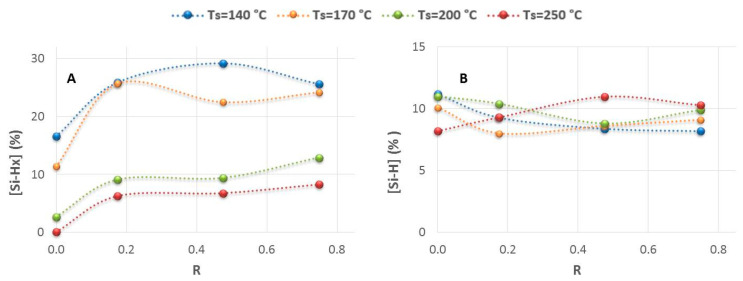
Evolution of atomic percentage of polyhydride [Si-H_x_] (**Graph A**) concentrations and monohydrides [Si-H] (**Graph B**) concentrations, as a function of the ratio R = Φ(PH_3_/H_2_)/Φ(SiH_4_), for the range of temperatures studied.

Figure 2 shows the evolution of hydrogen concentration arranged as polyhydrides (Si-H_x_) and monohydride (Si-H) bonds, as a function of the ratio R, for the temperature series. As can be seen in Figure 2A (left), the introduction of a low Φ (PH_3_/H_2_) (R = 0.18)) induces a significant increase in hydrogen content in the form of polyhydrides. This is particularly relevant in the sample deposited at 250 °C, which presents an almost negligible value of polyhydride concentration (0.1%) when it is prepared without phosphine. With the introduction of higher Φ(PH_3_/H_2_) than 3.5 sccm (R ≥ 0.48), a slight monotonic increase in polyhydrides is observed. In contrast, the variations in the hydrogen bonded as isolated monohydrides (Si-H) are very small and do not show a clear trend (Figure 2B, right), remaining practically unchanged, with atomic values between about 8.0 and 11.0%. It is worth noting the great differences in the polyhydride percentages between the series of high temperatures (T ≥ 200 °C) and low temperatures (T < 200 °C), which places temperature as one of the key deposition parameters for controlling the material composition and structure. It could be crucial to the behavior of electrodes in LIBs, as will be verified later.

**Figure 3:**
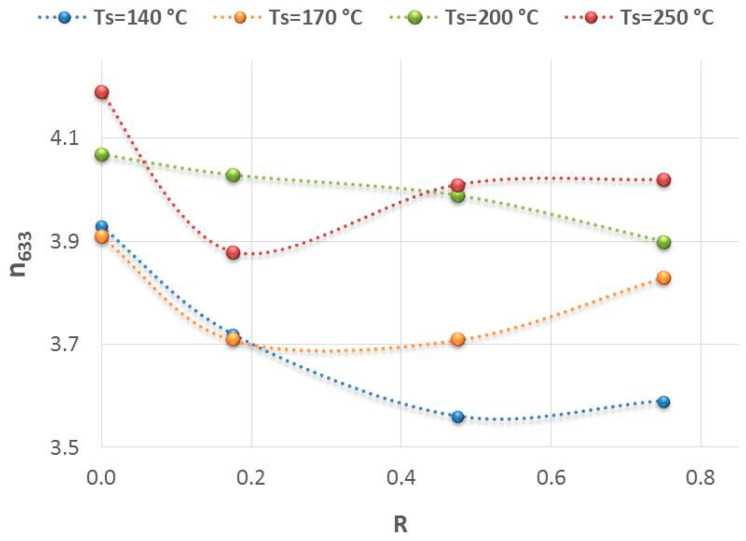
Evolution of the refractive index at 633 nm, as a function of the ratio R for the range of substrate temperatures studied.

It is also interesting to analyze the behavior of the refractive index as an indirect estimation of the mass density of a material [28,29]. As can be seen in Figure 3, the samples prepared at lower temperatures (T < 200 °C) exhibit lower refractive indexes associated with a less dense material, and a faster rate of decrease in the refractive index as the phosphine flow rate increases, indicating a greater loss of mass density. The marginal changes of n_633_ observed for the highest R = 0.75 at 140 °C and 170 °C could be attributed to the densification of material caused by the hydrogen gas. For this R value, the hydrogen flow rate from the mixture PH_3_/H_2_ becomes comparable to that of silane; that is, Φ(H_2_) = 14.7 sccm and Φ(SiH_4_) = 20 sccm, which produces a dilution effect that is not negligible.

**Figure 4:**
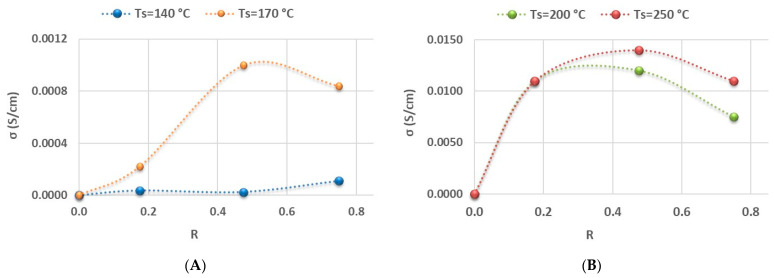
The evolution of electrical conductivity σ as a function of the ratio R is shown on (**Graph A**) for low deposition temperatures and on (**Graph B**) for high deposition temperatures.

Regarding the electrical properties of aSi:H films (Figure 4), the conductivity improved significantly when phosphine was incorporated as a dopant. The doping of the material can be changed from intrinsic to n-type by simply introducing 3.5 sccm of phosphine (low R = 0.18) into the gas mixture. Again, there are large differences of two orders of magnitude in the conductivity between samples deposited at low temperatures (Figure 4A) and high temperatures (Figure 4B).

**Figure 5:**
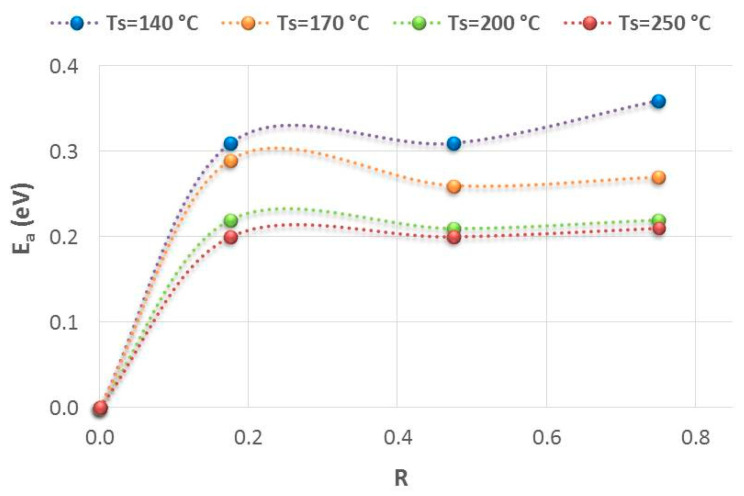
Evolution of activation energy as a function of the relation between phosphine flow rate and silane flow rate, R, for the different temperatures tested. No important changes have been observed with the increase in the concentration of phosphine or R for all deposition temperatures.

Furthermore, we have observed that for all the temperatures analyzed, the use of Φ (PH_3_/H_2_) greater than 3.5 sccm (R > 0.18) barely influences the doping level, since the activation energy (E_a_) remains practically invariable despite changes in the phosphine flow rate (see Figure 5). Therefore, under the deposition conditions studied, it is not necessary to introduce a high concentration of PH_3_ to improve the electrical properties. On the contrary, higher flow rates could give rise to a more defective and less compact material. It is possible that the excess phosphorous atoms are not incorporated as dopants, provoking defects in the structure such as dangling bonds and microvoids. Hydrogen atoms can passivate these defect states, which probably caused the observed increase in the hydrogen content, especially in polyhydride bonds. On the other hand, it seems that a better-quality material, with higher refractive indexes, can be more efficiently doped. This would explain the high σ achieved at deposition temperatures of 250 °C and 200 °C compared to those at lower temperatures (i.e., at 170 °C and 140 °C).

### 3.2. Assembly of aSi:H Films in Lithium-Ion Batteries

From the above results and observed trends, we have selected intrinsic and n-doped samples with different electrical conductivities and diverse polyhydride contents in order to analyze the influence of both electrical and structural characteristic of the aSi:H films integrated as electrodes on the behavior of LIBs, as can be seen in the forthcoming subsections. Table 1 summarizes the most relevant deposition conditions and properties of the electrodes used for this study.

#### 3.2.1. Effect of Electrical Properties on the Specific Capacity and Capacity Retention of LIBs

To determine the effect of electrical conductivity (σ) and activation energy (Ea) of aSi:H films on the behavior of the batteries during cycling, we have used non-doped samples (A&C), samples doped with very different conductivities (B&E), and samples doped with similar conductivities (B&D). In this section, we have focused solely on the material electrical properties in order to try to evaluate their effects in isolation, while remaining aware that the inclusion of dopant elements and the preparation conditions of the films will produce changes in the composition and structure, as previously discussed.

**Figure 6:**
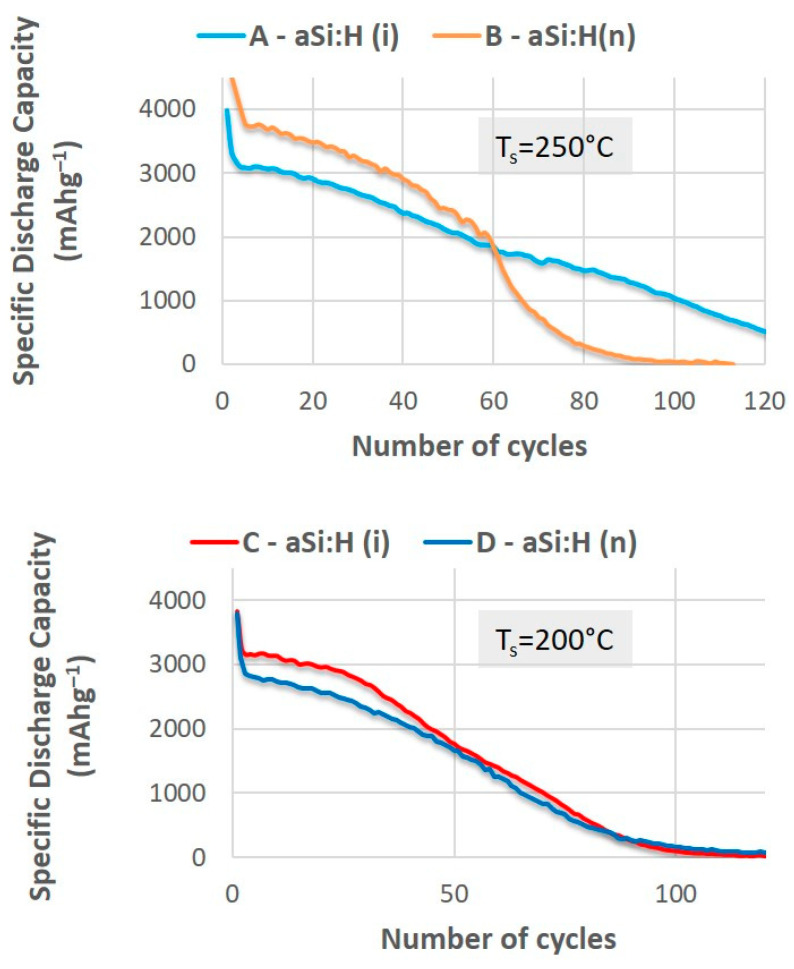
Galvanostatic curves of discharge capacity at the current density 1 Ag^−1^ versus number of cycles with intrinsic and n-doped a-Si:H anodes prepared at 250 °C and at 200 °C deposition temperatures.

Firstly, we compare pairs of batteries with aSi:H electrodes (doped (n) and intrinsic (i)) deposited at different temperatures (250 °C and 200 °C). In the batteries with electrodes deposited at 250 °C, shown in Figure 6, a significantly higher discharge capacity can be observed in the first thirty cycles for the conductive sample (battery B). However, the specific capacity of this battery decreases rapidly as the number of cycles increases and falls off sharply around 60 cycles, leading to total failure of the battery at 100 cycles, unlike the sample with the undoped amorphous silicon electrode (battery A), which keeps cycling for a greater number of cycles. In contrast, the batteries with both doped and intrinsic aSi:H electrodes, which were prepared at a lower temperature (batteries C and D at T_S_ = 200 °C, in Figure 6) show similar behaviors during cycling despite their different electrical properties, and have a durability comparable to that of battery B.

**Figure 7:**
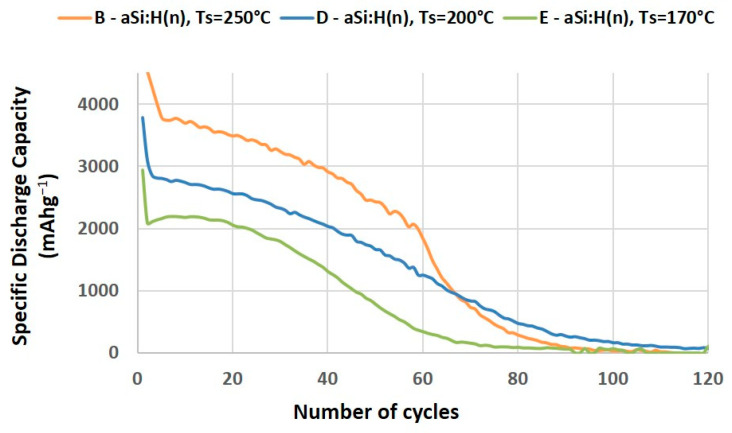
Specific discharge capacity curves of LIBs with doped a-Si:H electrodes with different preparation conditions.

If we also compare the batteries with n-type doped anodes that have practically identical conductivities and activation energies (B&D), regardless of the preparation conditions (Figure 7), we observe significant differences in their discharge-capacity evolution. This behavior strongly suggests that the nature and composition of the material impose more restrictive limitations on battery performance than electrical properties. Specifically, as shown in Figure 7, the doped electrode (E) exhibits a composition that is significantly different from the others, resulting in even more pronounced differences in specific capacity.

Based on the comparative analysis of these curves and the preceding discussion, it can be concluded that, for the aSi:H material examined in this investigation, enhanced electrical conductivity does not appear to be a predominant factor impacting battery performance. Instead, the compositional and structural variations arising from the incorporation of phosphorous atoms and the film deposition temperature could account for the faster rate of specific capacity decay observed in some batteries featuring n-doped electrodes.

#### 3.2.2. Effect of the Structural Properties (Si-H_x_ Concentration and Refractive Index) on Specific Capacity and Capacity Retention

After concluding that the electrical properties of the studied aSi:H films did not strongly affect the cycling performance of the batteries, and that this behavior could instead be conditioned by the nature of the aSi:H material, in this section, we analyze the influence of the structure and composition of the electrodes on the batteries. As mentioned in previous sections, the quality of hydrogenated amorphous silicon material in terms of its porosity and number of defects is well defined by the proportion of Si-H_x_ bonds, which are mainly arranged as clusters attached to the inner walls of microcavities, voids and pores in its bulk. Furthermore, the refractive index is related to the mass density of the material, and a decrease in its value reveals a loss of compactness. To evaluate this, we tested aSi:H films with different polyhydride contents and refractive indexes, regardless of the character material (n-type doped or intrinsic), as electrodes in LIBs.

**Figure 8:**
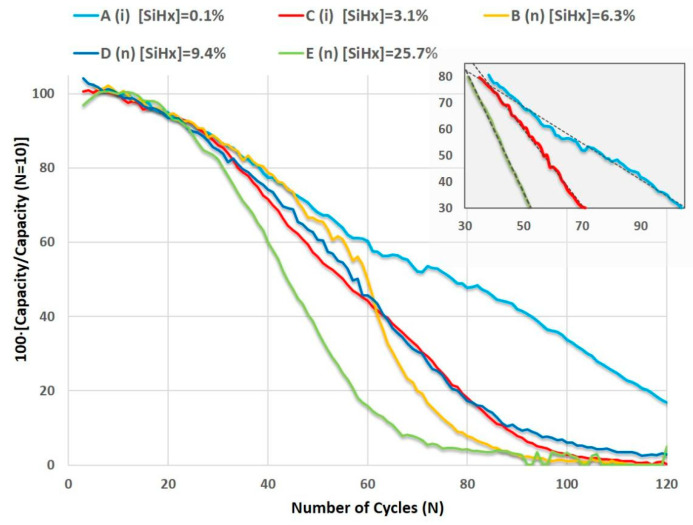
Normalized galvanostatic curves of the specific capacity of the LIBs with different, intrinsic (i) and doped (n), aSi:H electrodes. The polyhydride concentrations, [SiH_x_], the doped character of the a-Si:H electrodes and the lineal fit of three of the curves in the interval between 80% and 30% are also shown (embedded graph).

It is convenient to indicate that the low values of the active masses of the anodes could result in errors in the determination of the specific capacities of the LIBs, making it difficult to draw conclusive comparisons. Therefore, in order to analyze the differences between batteries and evaluate the stability based on the structural characteristics of the studied aSi:H anodes, we opted to assess the shapes of the galvanostatic curves in this section, as they better define the behavior and stability of the batteries with cycling. To achieve this, we normalized the discharge capacity curves of the tested LIBs, considering that the maximum initial capacity was obtained in the 10th cycle. Moreover, we conducted linear fitting of the normalized curves of discharge capacity within the range of 80% to 30% of the maximum capacity (Figure 8 and inset linear fitting).

**Figure 9:**
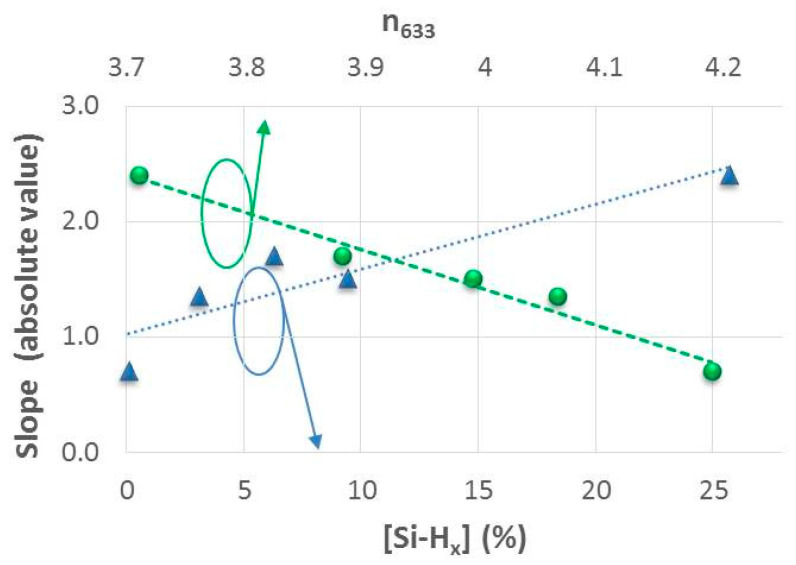
Slope absolute values of the lineal fits versus polyhydride concentration and refractive index (at 633 nm) for the LIBs previously represented in Figure 8. The dashed lines are a guide for the eyes.

The slopes calculated from the fits shown in Figure 8 were used as a guideline to compare the stability of batteries or the rate of battery decay based on the electrode properties. The slope (absolute value) clearly increases when a higher concentration of Si-H_x_ bonds is present in the amorphous silicon films, and in turn, the slope decreases for those electrodes with films that have a higher refractive index (Figure 9). These results indicate that the decrease in battery capacity during cycling is strongly influenced by the degree and type of hydrogenation, as well as the mass density of the aSi:H material. For this reason, the battery with an aSi:H anode that has a high concentration of [Si-H_x_], with R_SM_ ratio of 0.62 and high porosity (battery E), exhibits a 30% loss of its initial capacity in just 50 cycles. Conversely the battery with a denser anode that has an R_SM_ ratio of 0.01, which has an almost insignificant percentage of H attached as Si-H_x_ (battery A), retains its capacity up to 30% of the initial capacity beyond 100 cycles (as Figure 8 illustrates). Therefore, the amorphous structure of the more compact aSi:H film tested in these LIBs allows it to withstand the volume changes derived from the charge and discharge processes. Hence, the deposition conditions for these types of silicon-based electrodes grown using PECVD must be carefully controlled and fine-tuned to obtain denser and more conductive films that provide the advantage of better electrical properties without affecting the durability of the batteries.

**Figure 10:**
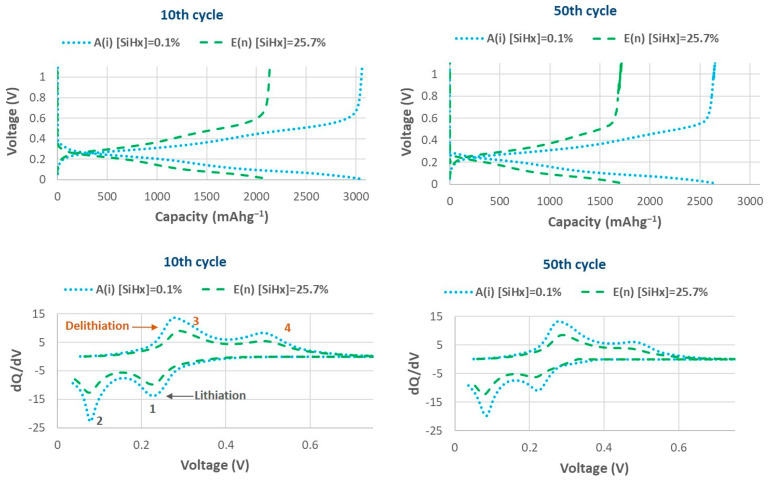
Galvanostatic charge–discharge curves (GCD) (**top graphs**) and differential capacity (dQ/dV) curves extracted from GCD profiles (**bottom graphs**), for different LIBs (polyhydride percentage of electrodes and the intrinsic or n-doped character is labeled) for the 5th (**left column**) and 50th (**right column**) cycles.

In order to compare the electrochemical results of batteries with a similar active mass and a different polyhydride concentration [Si-H_x_] in their electrodes, the galvanostatic charge–discharge curves (GCD), together with the resulting differential capacitance (dQ/dV) curves extracted from these GCD profiles for the 5th and the 50th cycles, are presented in Figure 10. The full range of [Si-H_x_] percentages is covered with data for batteries A and E, with [Si-H_x_] of 0.1% and 25.7%, respectively.

In the dQ/dV curves there are two relatively broad peaks (peaks 1 and 2 in Figure 10) corresponding to the lithiation processes, indicating single-phase transition reactions, since silicon is amorphous and transforms into amorphous phase as well. The exact compounds formed during the lithiation cycle are not well known, but it is assumed that there are two consecutive incorporations of Li into amorphous Si-Li phases [28], following the reactions below, where *x* ≈ 2.5 and *x* + *y* ≈ 3.5 (Equation (3)):

(3) $$\;aSi+xLi⇆a{Li}_{x}Si\;\;a{Li}_{x}Si+yLi⇆a{Li}_{x+y}Si$$

Regarding peaks 3 and 4, in dQ/dV curves, they correspond to the reverse reactions in the delithiation process. During delithiation, in all differential capacitance curves, single-phase transition peaks are observed. Peaks 3 and 4 are opposite to peaks 1 and 2, which means that delithiation, in the same way as lithiation, occurs between aSi and aLi_x+y_Si in two steps, each of which corresponds to one of the peaks. These two peaks appear in all cycles (see the 5th and 50th cycles as examples in Figure 10), indicating that these reactions are reversible. It is worth mentioning that battery E, which has a n-doped electrode with the highest polyhydride concentration (25.7%) shows the anodic peak 3 (Figure 10) at a slightly higher voltage, the so-called overpotential [30], than battery A, which has a non-doped electrode, despite the fact that its conductivity is significantly higher. This indicates that a relatively high anodic overpotential could be induced when the polyhydride concentration is very high. An overpotential usually suggests a higher internal resistance at the electrode arising from poor electrical contact or diffusion kinetics of the material [31] [32]. To corroborate the reasoning, we can observe the intensity of the peaks. The enclosed area under the dQ/dV curves specifies the capacity of the battery.

Conclusively, Figure 10 illustrates that the capacity of the batteries is mainly influenced by the material microstructure, so batteries with the lowest percentage of [Si-H_x_] present the highest capacity, with a greater area at their peaks. Based on these observations, we can deduce that electrodes formed from aSi:H films with low polyhydride content and a higher refractive index, that is denser and less defective, result in better battery performance, despite being non-conductive.

## 4. Conclusions

We have carried out an analysis of the electrical and compositional characteristics of non-nanostructured aSi:H thin films deposited via the PECVD technique with varying phosphine flow rates at different substrate temperatures. Our observations showed that the use of high phosphine flow rates did not further improve the electrical properties and instead increased the polyhydride percentages and decreased the refractive indexes of the films. These changes in optical properties and composition are usually attributed to more defective and porous material. By adjusting the substrate temperature, we were able to control the polyhydride contents, which allowed us to obtain n-doped aSi:H films with good electrical properties and acceptable quality.

Taking these premises into consideration, we tested non-conductive and conductive aSi:H anodes with different electrical properties and polyhydride contents. We observed that there is a linear correlation between the polyhydride concentration and the refractive index of the electrodes and the stability of the batteries. Consequently, we established that the degree and type of hydrogenation, and the mass density of the aSi:H material, are limiting factors for capacity retention. Our results showed that we were able to achieve LIBs with initial specific capacities of 3070 mAhg^−1^ (at 10th cycle), which can be retained at values higher than those of graphite for a significant number of cycles (more than 120 cycles) by controlling the structural properties of the aSi:H films. Additionally, electrodes formed from aSi:H films with low polyhydride content exhibit improved the battery performance. Therefore, hydrogenated amorphous silicon thin-films have demonstrated their suitability as an alternative for anodes in lithium-ion batteries. Our findings highlight that the PECVD technique offers the potential to explore various preparation conditions that can produce aSi:H films with high conductivities and low polyhydride contents. This would lead to a material with excellent properties for electrodes in LIBs.

## Figures and Tables

**Table 1:** Deposition parameters and optical, structural and electrical properties of aSi:H films deposited by PECVD that were selected as electrodes for LIBs. The silane flow rate was 20 sccm for all of them.

Sample	T_S_ (°C)	Φ(PH_3_/H_2_) (sccm)	R	m_act._ ± 0.05 (mg)	n_633_	[Si-H] (%)	[Si-H_x_] (%)	R_SM_	σ (S/cm)	E_a_ (eV)
A	250	0	0	0.37	4.19	7.4	0.1	0.01	4.0 × 10^−10^	--
B	250	3.5	0.18	0.40	3.88	9.3	6.3	0.21	1.1 × 10^−2^	0.20
C	200	0	0	0.34	4.06	8.8	3.1	0.12	4.0 × 10^−10^	--
D	200	9.5	0.48	0.43	3.99	8.8	9.4	0.31	1.2 × 10^−2^	0.20
E	170	3.5	0.18	0.37	3.71	8.0	25.7	0.62	2.2 × 10^−4^	0.29

## Data Availability

Data are contained within the article.
